# Prolonged impairment of immunological memory after anti-CD20 treatment in pediatric idiopathic nephrotic syndrome: an extended follow-up

**DOI:** 10.3389/fimmu.2025.1736921

**Published:** 2025-12-18

**Authors:** Manuela Colucci, Martina Riganati, Federica Zotta, Antonio Gargiulo, Laura Massella, Barbara Ruggiero, Nicola Cotugno, Giulia Ricci, Paolo Palma, Francesco Emma, Marina Vivarelli

**Affiliations:** 1 Nephrology Research Unit, Bambino Gesù Children’s Hospital, Istituto Di Ricovero e Cura a Carattere Scientifico (IRCCS), Rome, Italy; 2 Division of Nephrology, Bambino Gesù Children’s Hospital, Istituto Di Ricovero e Cura a Carattere Scientifico (IRCCS), Rome, Italy; 3 Unit of Clinical Immunology and Vaccinology, Bambino Gesù Children’s Hospital, Istituto Di Ricovero e Cura a Carattere Scientifico (IRCCS), Rome, Italy; 4 Department of Systems Medicine, University of Rome “Tor Vergata”, Rome, Italy

**Keywords:** anti-CD20 treatment, B cells, hypogammaglobulinaemia, idiopathic nephrotic syndrome (INS), immunological memory, pediatric nephrology

## Abstract

**Introduction:**

Anti-CD20 therapy is an effective steroid-sparing option for pediatric idiopathic nephrotic syndrome (INS), but long-term data on immune reconstitution are limited.

**Methods:**

Thirteen pediatric INS patients (7 males) were longitudinally evaluated at baseline, first long-term follow-up (mean 5.4 years), and extended follow-up (mean 6.6 years after the first follow-up, >3 years from the last anti-CD20 infusion). Clinical outcomes, B-cell subsets, serum immunoglobulin levels, vaccine competence, and infection rates were analyzed.

**Results:**

At the first follow-up, most patients had received one (n=6) or two (n=6) anti-CD20 courses; at the extended follow-up, five had undergone additional treatments. Four patients remained relapse-free during follow-up, whereas eight of nine who had previously relapsed continued to experience disease recurrence despite further anti-CD20 therapy. Oral immunosuppressant tapering improved: three patients were off therapy at first follow-up and six at the latest. Total, transitional and mature-naïve B cells reconstituted to normal ranges according to age over time. In contrast, total, IgM, and switched memory B cells remained significantly reduced (p<0.01). Patients in sustained remission exhibited lower switched memory B-cell counts than relapsing patients (p<0.05). Serum IgG levels increased at the extended follow-up, although six patients remained below normal. Four developed severe *de novo* hypogammaglobulinemia requiring long-term immunoglobulin replacement and showing increased infection susceptibility. Vaccine-specific IgG titers against tetanus and HBV remained below the limit of seroprotection despite re-immunization in most patients.

**Conclusions:**

Anti-CD20 therapy offers durable disease control and allows immunosuppressant reduction in pediatric INS, but persistent memory B-cell and humoral impairment warrant long-term immunologic monitoring.

## Introduction

B-cell depletion by anti-CD20 therapy has become an established therapeutic option for pediatric idiopathic nephrotic syndrome (INS), mainly for patients who experience frequent relapses and/or steroid-dependence (1–3). However, accumulating evidence indicates that such treatment profoundly alters B-cell homeostasis and long-term humoral immunity in this population. Previous studies have consistently shown that a substantial proportion of pediatric INS patients - approximately 40-60% - develop hypogammaglobulinemia following anti-CD20 therapy, sometimes severe and persisting beyond one year (4–10). The development of therapy-induced hypogammaglobulinemia appears to be independent of ethnicity but is strongly associated with younger age at first anti-CD20 infusion, steroid-resistant disease, and low baseline IgG levels (4–7, 9, 11). Although total B cells generally recover within six months, memory and switched-memory B-cell subsets may remain significantly reduced for over a year, often paralleling delayed IgG normalization (4). Impaired vaccine responses have also been described, with decreased anti-hepatitis B virus (HBV) and anti-tetanus antibody titers and suboptimal IgG levels even after re-immunization (4, 12). Despite these insights, data on the long-term immunological outcomes of pediatric INS patients treated with anti-CD20 therapy - particularly beyond two years after treatment - remain scarce. The persistence or resolution of immunological memory defects over extended follow-up periods has not been systematically investigated. The present brief report provides an extended long-term follow-up of our previously described pediatric INS cohort treated with anti-CD20 therapy (4), focusing on the durability of immunological alterations, including B-cell subset distribution and serum immunoglobulin levels, several years after treatment.

## Methods

### Study population

**Figure 1:**
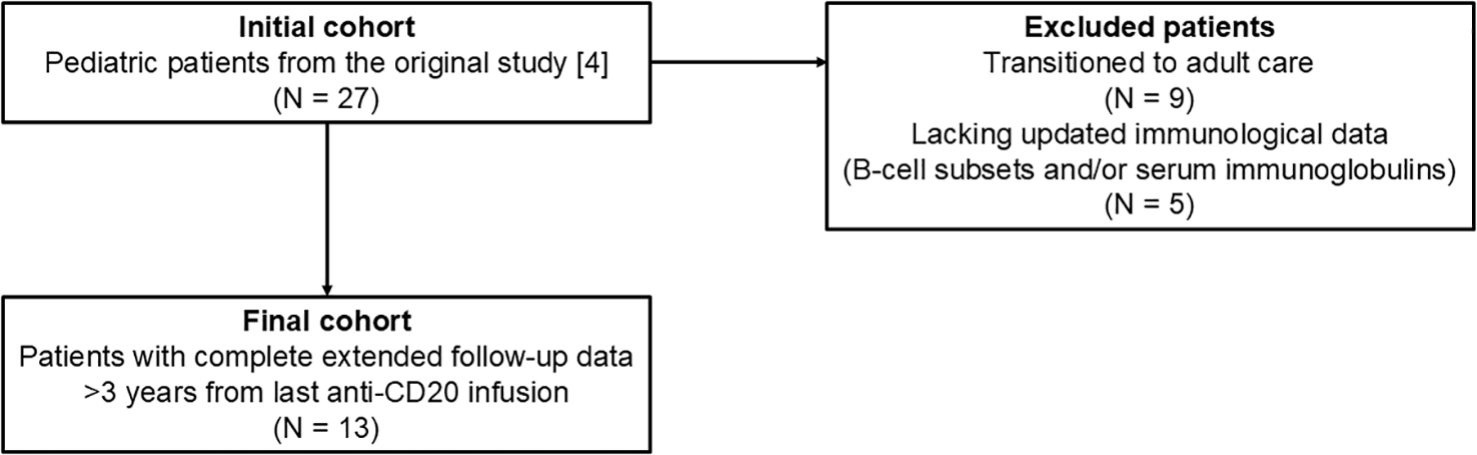
Patient study flowchart.

This study is a long-term observational follow-up of a previously published pediatric cohort with INS treated with anti-CD20 therapy (rituximab or ofatumumab) at Ospedale Pediatrico Bambino Gesù. Inclusion criteria were as follows: frequently-relapsing (defined as ≥2 consecutive relapses within 6 months or ≥3 relapses within any 12-month period) or steroid-dependent (defined as ≥2 consecutive relapses during prednisone treatment or within 14 days of its discontinuation) nephrotic syndrome (13); a minimum follow-up >3 years from the last anti-CD20 infusion; available data on circulating B-cell subsets and on serum immunoglobulin levels. Among the 27 patients included in the original study, patients who had transitioned to adult care (n = 9) or lacked updated data on B-cell subsets or serum immunoglobulin levels (n = 5) were excluded. The final analysis comprised 13 patients with complete longitudinal data (**Figure 1**).

All patients had steroid-dependent nephrotic syndrome. Relapse was defined as proteinuria ≥ 3+ for at least three consecutive days on urine dipstick testing (13).

Patients received a single infusion of anti-CD20 therapy, followed by a second infusion administered seven days later in cases of incomplete B-cell depletion (CD19^+^ B cells > 10 cells/μL of peripheral blood lymphocytes). All patients initially received rituximab (375 mg/m²). Among those who underwent multiple infusions (≥ 2), two patients received ofatumumab (1500 mg/1.73 m²) as their final anti-CD20 treatment.

Anti-CD20 therapy was administered during steroid-induced remission, and repeat courses were given only in the event of relapse, except for two patients who experienced rapid B-cell recovery (within 1 and 3 months, respectively). Demographic and clinical characteristics, number of relapses, infectious episodes, concurrent immunosuppressive therapies (prednisone, mycophenolate mofetil, and calcineurin inhibitors), number and type of anti-CD20 infusions, and use of immunoglobulin replacement therapy were recorded. Concomitant immunosuppressive agents were tapered or discontinued following anti-CD20 administration unless relapse occurred.

### Sample procurement and cell isolation

Blood samples were collected in accordance with institutional guidelines for informed consent, following approval by the local Ethics Committee and in compliance with the Declaration of Helsinki. Samples were obtained at baseline (time of the first anti-CD20 infusion), 2–7 days post-infusion, at first follow-up (>2 years after the last infusion), and at extended follow-up (>3 years after the last infusion). Peripheral blood mononuclear cells (PBMCs) were isolated using Ficoll-Paque Plus (Amersham Biosciences) density-gradient centrifugation.

### Flow cytometry

PBMCs were stained with fluorochrome-conjugated monoclonal antibodies targeting CD19, CD24, CD27, CD38, IgD, and IgM (BD Biosciences) and analyzed by multicolor flow cytometry (FACS BD LSRFortessa, BD Biosciences) (14). Subsets of gated CD19^+^ B cells were identified based on surface marker expression as follows: transitional (CD38^high^CD24^high^) and mature-naïve (CD38^intermediate^CD24^low^) B cells, expressed as absolute counts. Memory B cells were defined as CD19^+^CD27^+^ cells, and memory subsets were classified as IgM memory (IgM^+^IgD^intermediate^) or switched memory (IgM^-^IgD^-^), as previously described (14). Data was analyzed using FACSDiva software, with 50,000 live lymphocyte events gated per sample. Age-related reference ranges were based on previously published data (15).

### Immunoglobulin levels

Total serum IgG, IgA, and IgM concentrations, as well as anti-tetanus and anti-HBV IgG titers, were measured as part of routine diagnostics in the clinical laboratory of the Ospedale Pediatrico Bambino Gesù. Immunoglobulin deficiency detected at the last follow-up but absent at baseline was classified as “*de novo”*.

## Results

### Study patients

Clinical characteristics of the 13 included patients (7 males) are summarized in **Table 1**. Data collected at baseline (time of first anti-CD20 administration) and at the first long-term follow-up >2 years from last anti-CD20 infusion (mean: 5.4 years; range: 2.1–7.1 years after anti-CD20 infusion) were previously reported in the original study (4). At the first follow-up, most patients had received one (n = 6) or two (n = 6) courses of anti-CD20 therapy, while one patient had received five courses.

**Table 1:** Characteristics of patients.

Parameter	Unit	Baseline (n = 13)	First follow-up (n=13)	Extended follow-up (n=13)
Demographics				
Age, mean [range]	Years	10.3 [5.1-14.8]	16.6 [9.6-21-8] ^c^	23.2 [15.9-29.6] ^c^
Male sex	N (%)	7 (54)	Same	Same
Anti-CD20 courses				
Course 1	N (%)	–	6 (46)	4 (31)
Course 2	N (%)	–	6 (46)	5 (38)
Course 3	N (%)	–	0 (0)	2 (15)
Course 5	N (%)	–	1 (8)	2 (15)
Post-anti-CD20 relapse	N (%)	–	9 (69)	9 (69)
Oral immunosuppressive drugs in the previous 12 months				
Prednisone alone	N (%)	0 (0)	0 (0)	1 (8)
MMF alone	N (%)	0 (0)	5 (38) *	4 (31)
Prednisone + MMF	N (%)	3 (23)	2 (15)	2 (15)
Prednisone + CNIs	N (%)	5 (38)	1 (8)	0 (0) *
Prednisone + MMF + CNIs	N (%)	6 (46)	2 (15)	0 (0) *
No immunosuppressive drug	N (%)	0 (0)	3 (23)	6 (46) *^†^
B cell subsets below the age-related normal range^a^				
CD19 positive	N (%)	5 (42) ^d^	0 (0)	1 (8) *
Transitional	N (%)	11 (92) ^d^	0 (0) ***	0 (0) ***
Mature	N (%)	6 (50) ^d^	0 (0) **	1 (8) *
Memory	N (%)	2 (17) ^d^	9 (69) *	8 (62) *
IgM memory	N (%)	2 (17) ^d^	3 (23)	7 (54)
Switched memory	N (%)	2 (17) ^d^	11 (85) **	10 (77) *
Immunoglobulins below the normal range^b^				
IgG	N (%)	7 (54)	10 (77)	6 (46) ^e^
Severe hypo-IgG	N (%)	0 (0)	4 (31)	0 (0) ^e^
IgA	N (%)	2 (15)	5 (38)	7 (54) ^e^
Severe hypo-IgA	N (%)	0 (0)	4 (31)	5 (38) ^e,^*
IgM	N (%)	2 (15)	0 (0)	1 (8) ^e^
Need for Ig replacement	N (%)	0 (0)	4 (31)	4 (31)
Impaired antigen-specific IgG titer ^b^				
Anti-HBV < 10 mIU/ml	N (%)	9 (75) ^d^	12 (92)	8 (62) ^e^
Anti-tetanus < 0.1 IU/ml	N (%)	5 (42) ^d^	8 (62)	4 (31) ^e^
Anti-tetanus < 0.6 IU/ml	N (%)	12 (100) ^d^	13 (100)	8 (62) ^e,^*^,†^
Infections	N (%)	–	4 (31)	7 (54)

Continuous data are expressed as mean and range, compared using unpaired t-test or one way ANOVA, and pairwise comparisons were evaluated by a Bonferroni *post-hoc* analysis. Categorical values are indicated as absolute count and percentage, compared by a Fisher’s exact test.

MMF, mofetil myocophenolate; CNIs, calcineurin inhibitors. ^a^ Range indicated in (15). ^b^ Range indicated in the diagnostic laboratory of our Institution. ^c^ Age differences reflect the time elapsed between study timepoints. ^d^ N=12. ^e^ N = 4 patients were receiving Ig replacement at this timepoint.

*, p<0.05, **, p<0.01, ***, p<0.001, *vs* Baseline. ^†^, p<0.05 *vs* First Follow-up.

At the extended follow-up (mean: 6.6 years after the previous evaluation), two patients had received a second anti-CD20 retreatment, two a third, and another a fourth and fifth retreatment, all administered in response to disease relapse (**Table 1**). The extended follow-up included data collected >3 years after the last anti-CD20 infusion (range: 3.2–16.0 years) and more than 9 years after the first infusion (range: 9.0–16.7 years).

From a clinical perspective, four patients who were already in sustained remission at the first long-term follow-up remained relapse-free throughout the extended follow-up. In contrast, eight of nine patients who had relapsed by the first follow-up continued to experience relapses despite additional anti-CD20 courses. However, one patient who had a single relapse one year after the first anti-CD20 course subsequently achieved long-term remission, which persisted without further anti-CD20 treatment up to the extended follow-up (6.6 years after the previous evaluation).

Importantly, median duration of concomitant oral immunosuppression after first anti-CD20 infusion was 10 years (range 1–14 years) and a progressive reduction in oral immunosuppressive therapy was observed over time. At the first long-term follow-up, three patients had successfully discontinued all immunosuppressive agents. This improvement became even more pronounced at the extended follow-up, when six patients were completely off immunosuppressive therapy for more than 3 years since the discontinuation of the last immunosuppressive drug (range: 3–15 years). Moreover, none of the remaining patients required triple therapy or the combination of prednisone with calcineurin inhibitors, and only a few continued on single or dual regimens with prednisone (n = 1), mycophenolate mofetil (n = 4), or their combination (n = 2) (**Table 1**).

### B-cell subset recovery

**Figure 2:**
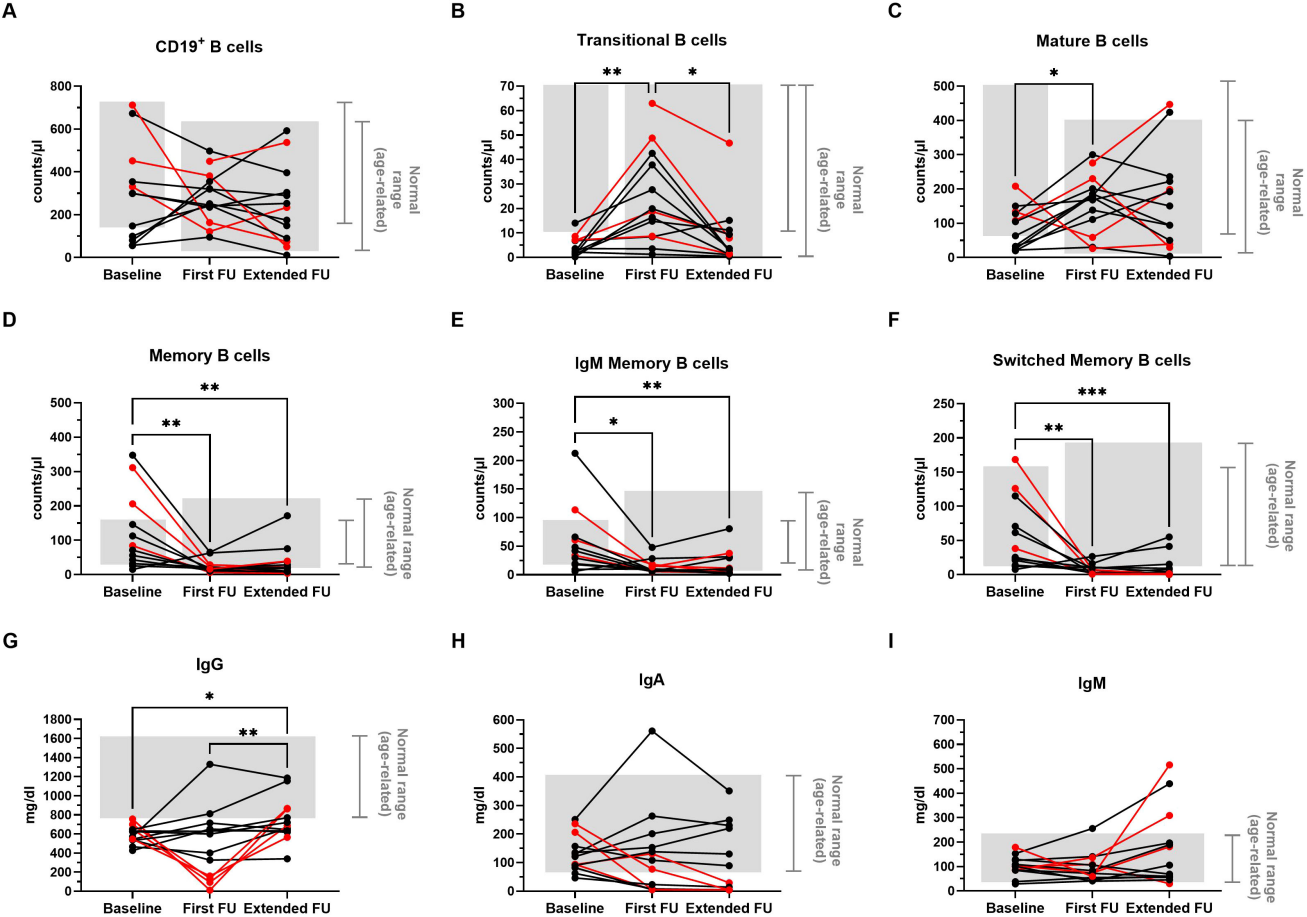
Extended long-term effects of anti-CD20 treatment on circulating B-cell subsets and total serum immunoglobulin levels in children with idiopathic nephrotic syndrome (INS). Levels of B-cell subsets **(A–F)** and immunoglobulins **(G–I)** were analyzed in pediatric INS patients (n = 13) before treatment (Baseline), after more than 2 years (First long-term follow-up, FU), and more than 3 years after the last anti-CD20 infusion (Extended FU). **(A–F) (A)** Total CD19^+^ B cells were identified based on surface marker expression as previously described (4). **(B)** Transitional, **(C)** mature-naïve, **(D)** total memory, **(E)** IgM memory, and **(F)** switched memory B cells are expressed as absolute cell counts per microliter of blood. **(G–I) (G)** Total IgG, **(H)** IgA, and **(I)** IgM levels are expressed in mg/dL. Each plot represents a different patient. Gray areas indicate the age-related normal range, as reported by Blanco et al. for B-cell subsets (15) and by the diagnostic laboratory of our institution for immunoglobulins. The ranges differ among timepoints due to the time elapsed between study assessments. Red lines and dots identify patients who developed *de novo* hypogammaglobulinemia at the first FU and required initiation of immunoglobulin replacement therapy. Differences between groups were compared using the nonparametric Kruskal–Wallis test; when significant, pairwise comparisons were performed using Dunn’s multiple comparisons test. *, p<0.05; **, p<0.01; ***p<0.001.

Longitudinal changes in circulating B-cell subsets are shown in **Figure 2**. Normalization of total CD19^+^ and mature naïve B-cell counts was observed at both the first and extended follow-ups (**Figures 2A, C**). Transitional B cells, which were significantly increased at the first long-term follow-up, returned to baseline levels - within age-related normal ranges - at the extended follow-up (**Figure 2B**). In contrast, the marked reduction in total memory, IgM memory, and switched memory B-cell counts observed at the first long-term follow-up persisted at the extended follow-up (**Figures 2D–F**).

Of note, among all B-cell subsets analyzed, patients who remained relapse-free after the last anti-CD20 course showed significantly lower median levels of switched memory B cells compared with relapsing patients (median [IQR]: 0.48 [0.13–2.16] vs. 7.68 [2.07–28.28], p < 0.05).

### Serum immunoglobulin levels

Longitudinal changes in serum immunoglobulin concentrations are shown in **Figures 2G–I**. Median serum IgG levels significantly increased at the extended follow-up (**Figure 2G**), although they remained below the normal range in six patients (**Table 1**). In contrast, serum IgA and IgM levels did not show significant changes throughout the entire follow-up period (**Figures 2H, I**). However, four patients developed *de novo* severe hypogammaglobulinemia (defined as serum IgG < 160 mg/dL), irrespective of the number of anti-CD20 treatment courses (one course in two patients, two in one patient, and five in one patient), detected already at first long-term follow-up. Two of these patients were the youngest and the only below 6 years old at the time of the first anti-CD20 infusion. All four patients required initiation of immunoglobulin replacement therapy (0.2-0.3 g/Kg every 2–4 weeks, adjusted based on clinical efficacy) (**Figure 2G**). Attempts to discontinue replacement therapy during the extended follow-up period were unsuccessful, due to marked decline in IgG levels and/or the occurrence of infections upon discontinuation of immunoglobulin replacement. Consequently, all four patients remained on immunoglobulin replacement therapy more than four years after its initiation (range: 4.5–10.4 years), which likely masked their true endogenous serum IgG concentrations at the extended follow-up (**Figure 2G**, **Table 1**).

Additionally, *de novo* severe hypo-IgA was detected in four patients at the first long-term follow-up (in association with severe hypo-IgG in two cases) and in one additional patient at the extended follow-up (**Figure 2H**, **Table 1**).

### Vaccine competence and infections

Despite vaccination against tetanus and HBV according to national immunization schedules before anti-CD20 administration, and re-immunization in seven patients after the last anti-CD20 infusion, most patients showed impaired vaccine competence based on vaccine-specific IgG titers at baseline and at the first long-term follow-up (**Table 1**). The significant increase in the number of patients protected against tetanus observed at the extended follow-up could be attributable to immunoglobulin replacement therapy in four patients, as described above for total IgG levels, and to re-immunization in one patient (**Table 1**).

Infectious events were reported in four patients at the first follow-up and in seven patients at the extended follow-up (**Table 1**). Among them, frequent (monthly during the winter season) respiratory tract infections were reported in four patients, and pneumonia and an HZV infection requiring hospitalization were reported in two different patients.

## Discussion

In this brief report, we provide an extended long-term follow-up of pediatric patients with INS treated with anti-CD20 therapy, extending our previous observations to more than nine years from the first exposure and over three years from the last infusion.

From a clinical perspective, our findings confirm that anti-CD20 therapy remains effective in reducing relapse rates and steroid dependence in children with INS. Anti-CD20 treatment enabled substantial tapering or discontinuation of oral immunosuppressive drugs, with several patients maintaining disease remission off combination therapy. This benefit may be partly attributable to the cumulative effect of repeated anti-CD20 infusions (five patients received one or two additional courses) and partly to the increasing age at each administration, which is known to reduce relapse risk in pediatric patients (11).

However, our data also demonstrates that anti-CD20 therapy induces alterations in B-cell homeostasis and humoral immunity, persistent even many years after treatment. Total CD19^+^ and mature naïve B cells normalized in all patients at both the first and extended long-term follow-ups, and transitional B cells returned to age-appropriate ranges, reflecting robust reconstitution of the overall B-cell compartment. In contrast, memory B cells - particularly switched memory subsets - remained significantly reduced in most patients, consistent with our previous findings and indicative of a prolonged impairment of immunological memory (4). Although concomitant immunosuppression may contribute to the prolonged immunological impairment, it cannot induce it on its own, since the impairment persists for several years after discontinuation of all immunosuppressive drugs in some patients in our cohort and is not caused by a combination of oral immunosuppressive drugs in a comparable group of pediatric INS patients who never received anti-CD20 therapy, as we previously reported (4). Interestingly, although the sample size was limited, patients who maintained long-term remission after their last anti-CD20 infusion seemed to exhibit lower switched memory B-cell counts compared to those who relapsed. These results mirror previous observations by our group and others, which reported that the recovery of memory B cells - and in particular of switched memory B cells - is associated with a higher risk of relapse following anti-CD20 therapy, further supporting a potential link between durable disease control and delayed reconstitution of antigen-experienced B cells (14, 16–18).

Serum immunoglobulin concentrations were generally stable, with IgG levels showing a tendency to improve at the extended follow-up, although normalization was not achieved in most patients. Furthermore, four patients developed a *de novo* severe hypogammaglobulinemia after anti-CD20 therapy, irrespective of the number of treatment courses, and required long-term immunoglobulin replacement. Attempts to discontinue replacement were unsuccessful, and all affected patients remained on immunoglobulin replacement therapy at the last follow-up. Of note, two of these patients were the youngest ones (<6 years old) at the time of first anti-CD20 exposure, consistent with previous reports suggesting that younger age at first infusion increases the risk of persistent hypogammaglobulinemia (4–6, 9, 11). Interestingly, as previously observed for reduced switched memory B cells, a trend toward fewer relapses was also reported among patients with prolonged post-anti-CD20 hypogammaglobulinemia (4, 6).

Vaccine-induced immunity was also affected, with low anti-tetanus and anti-HBV IgG titers at baseline showing only partial recovery following re-immunization at the first follow-up, evaluated before starting Ig replacement. The increased proportion of patients protected against tetanus at the extended follow-up is in part attributable to re-immunization (in one patient) and in part could be attributable to immunoglobulin replacement therapy rather than restoration of endogenous antibody production, since it is observed in all the patients who were receiving Ig replacement. Moreover, persistent susceptibility to infections, with hospitalization required in two patients, suggests that anti-CD20 therapy predisposes patients to a sustained increased risk of infection. These findings underscore the need for individualized strategies to preserve or restore vaccine competence in patients undergoing B-cell-depleting therapy.

The main limitations of the current study are the small sample size and its monocentric and retrospective design, which together do not allow any standardization of the treatment, either in terms of anti-CD20 therapy or concomitant oral immunosuppressive drugs. More importantly, the apparent high proportion of patients who experienced a severe hypogammaglobulinemia requiring prolonged Ig replacement in our cohort should not be generalized, since it is due to our stringent inclusion criterion of a long follow-up after the last anti-CD20 therapy. However, the need for Ig replacement following anti-CD20 treatment is not uncommon, as recently reported in a multicenter retrospective study investigating the therapeutic efficacy and adverse events associated with repeated anti-CD20 administrations in a large cohort of pediatric INS patients (11).

Overall, our results reinforce the disease-modifying potential of anti-CD20 therapy in pediatric INS. At the same time, they highlight an important trade-off between sustained disease control and long-term humoral immune impairment with potential implications for infectious risk and vaccination strategies, which must be carefully balanced, particularly in younger patients or those treated early in the disease course. Longitudinal monitoring of B-cell subsets, immunoglobulin levels, and vaccine responses should therefore be incorporated into the routine follow-up of pediatric INS patients treated with anti-CD20 therapy. These considerations are particularly relevant now that more potent B-cell–depleting agents, such as obinutuzumab, plasma cell-targeting therapies such as daratumumab, or anti-CD19/BCMA CAR-T cell therapies, are emerging as promising treatment options for severe or refractory cases (19–22).

## Data Availability

The raw data supporting the conclusions of this article will be made available by the authors, without undue reservation.
